# Ontogeny reversal and phylogenetic analysis of *Turritopsis* sp.5 (Cnidaria, Hydrozoa, Oceaniidae), a possible new species endemic to Xiamen, China

**DOI:** 10.7717/peerj.4225

**Published:** 2018-01-08

**Authors:** Jun-yuan Li, Dong-hui Guo, Peng-cheng Wu, Li-sheng He

**Affiliations:** 1 Department of Life Sciences, Institute of Deep-Sea Science and Engineering, Chinese Academy of Sciences, Sanya, Hainan, China; 2 College of Earth Sciences, University of Chinese Academy of Sciences, Beijing, China; 3 College of Ocean and Earth Sciences, Xiamen University, Xiamen, Fujian, China

**Keywords:** *Turritopsis*, Reverse development, Medusa, Hydroid, China

## Abstract

Ontogeny reversal, as seen in some cnidarians, is an unprecedented phenomenon in the animal kingdom involving reversal of the ordinary life cycle. Three species of *Turritopsis* have been shown to be capable of inverted metamorphosis, a process in which the pelagic medusa transforms back into a juvenile benthic polyp stage when faced with adverse conditions. *Turritopsis* sp.5 is a species of *Turritopsis* collected from Xiamen, China which presents a similar ability, being able to reverse its life cycle if injured by mechanical stress. Phylogenetic analysis based on both 16S rDNA and cytochrome c oxidase subunit I (*COI*) genetic barcodes shows that *Turritopsis* sp.5 is phylogenetically clustered in a clade separate from other species of *Turritopsis*. The genetic distance between *T*. sp.5 and the Japanese species *T*. sp.2 is the shortest, when measured by the Kimura 2-Parameter metric, and the distance to the New Zealand species *T. rubra* is the largest. An experimental assay on the induction of reverse development in this species was initiated by cutting medusae into upper and lower parts. We show, for the first time, that the two dissected parts have significantly different potentials to transform into polyps. Also, a series of morphological changes of the reversed life cycle can be recognised, including medusa stage, contraction stage I, contraction stage II, cyst, cyst with stolons, and polyp. The discovery of species capable of reverse ontogeny caused by unfavorable conditions adds to the available systems with which to study the cell types that contribute to the developmental reversal and the molecular mechanisms of the directional determination of ontogeny.

## Introduction

Reverse ontogeny is a phenomenon first discovered in *Chrysaora hysoscella* (as *C. mediterranea*, Scyphozoa) (Hadži, 1909). It involves ontogenetic reversal of the life cycle from one stage to the preceding stage (polyp stage for *C. hysoscella*), and it is induced by unfavorable conditions. This reversible life cycle could be considered to be a metamorphosis, involving the transformation of the morphology of an individual, but in the opposite direction to the usual developmental route. This is different from regeneration, which only reorganizes the original morph. Reverse development is not exclusive to scyphozoans; other cnidarian taxa also exhibit similar properties. In Anthozoa, *Pocillopora damicornis* L. polyps retract all tissues from corallites and transform into an initial planula-like stage when the polyps experience stress shortly after attaching to a substrate (Richmond, 1985). Some hydrozoan species have the ability to reverse their life cycles as well. Mechanically isolated, early stage medusa buds from gonozooids of *Podocoryna carnea* M. Sars can transform back into stolons or polyps (Frey, 1968; Müller, 1913; Schmid, 1972). Comparable phenomena are also observed in other hydrozoans, such as *Eleutheria dichotoma* Quatrefages (Hauenschild, 1956), *Cladonema* sp., *Cladonema uchidai* Hirai (Kakinuma, 1969), and *Laodicea undulate* Forbes and Goodsir (De Vito et al., 2006; Kubota, 2006). Since all of the above-mentioned species have the potential for reverse development, it has been suggested that ontogeny reversal is not a rare phenomena in cnidarians and might represent an adaptive response to adverse environments.

Ontogeny reversal is a complex process; tissue reorganization and substitution of cell types are involved. Studies on *P. carnea* indicate that both the proliferation of interstitial cells and the transdifferentiation of fully differentiated cells participate in this reversal (Alder & Schmid, 1987; Schmid, 1972; Schmid & Alder, 1984; Schmid, Wydler & Alder, 1982). Nevertheless, many points underlying this process—such as the mechanisms regulating the establishment and maintenance of the functional stability of cells or the cooperation of signal networks that control the direction of ontogeny—are still unclear. The discovery of a species that is capable of reverse development and the subsequent morphological characterization of different stages during the reverted life cycle provides the opportunity to further study these questions.

Reverse development in the previously-mentioned species in Cnidaria occurs only during the early stages of their development and is lost in older stages (Frey, 1968; Kubota, 2011; Müller, 1913; Piraino et al., 1996; Schmid, 1972). *Turritopsis dohrnii* Weismann, a hydrozoan species, breaks this rule, and shows that ontogeny reversal can occur at all stages of medusa growth, including the adult stage with mature gonads. Mature *T. dohrnii* can transform into stolons and polyps spontaneously even when cultured under favorable conditions (Kubota, 2011; Piraino et al., 1996, not *T.nutricula* see Schuchert, 2004). This implies that even if there is no extraneous stress inducing the reverse development, a genetically programmed endogenous factor could still be a trigger to the organism reversing its life cycle. When used in the study of reverse development, *T. dohrnii* might reveal more pathways related to activating life stage reversal than could be found from other cnidarians.

Up to now, only three *Turritopsis* species with life cycle reversal abilities have been reported: *T. dohrnii* (Kubota, 2011; Piraino et al., 1996), *T. rubra* Farquhar (Kubota, 2005)*,* and *T.* sp.2 (Kubota, 2005; Kubota, 2015), of which the last two were identified as *T. nutricula* McCrady with different medusa morphotypes by Kubota in 2005, but they were phylogenetically clustered as two separate clades from the *T. nutricula* clade based on molecular data in Miglietta’s work (Miglietta et al., 2007; Miglietta & Lessios, 2009). Several exogenous inductions have been conducted in order to activate the ontogeny reversal of *Turritopsis,* including starvation, temperature or salinity change, exposure to heavy metals, and mechanical injury. In China, *Turritopsis* are widely distributed in the bays of Xiamen, however, their classification status and capability to reverse the life cycle have not been well characterized. The present study reports on a population of *Turritopsis* (*T.* sp.5), which is a possibly new species endemic to China. *Turritopsis* sp.5 is phylogenetically clustered in a different clade from the three previously known species, but still has the ability to revert into the polyp stage after experiencing mechanical damage. The morphologies of each stage in the reversed life cycle are described, and the features of the process of reverse development are discussed.

**Figure 1:**
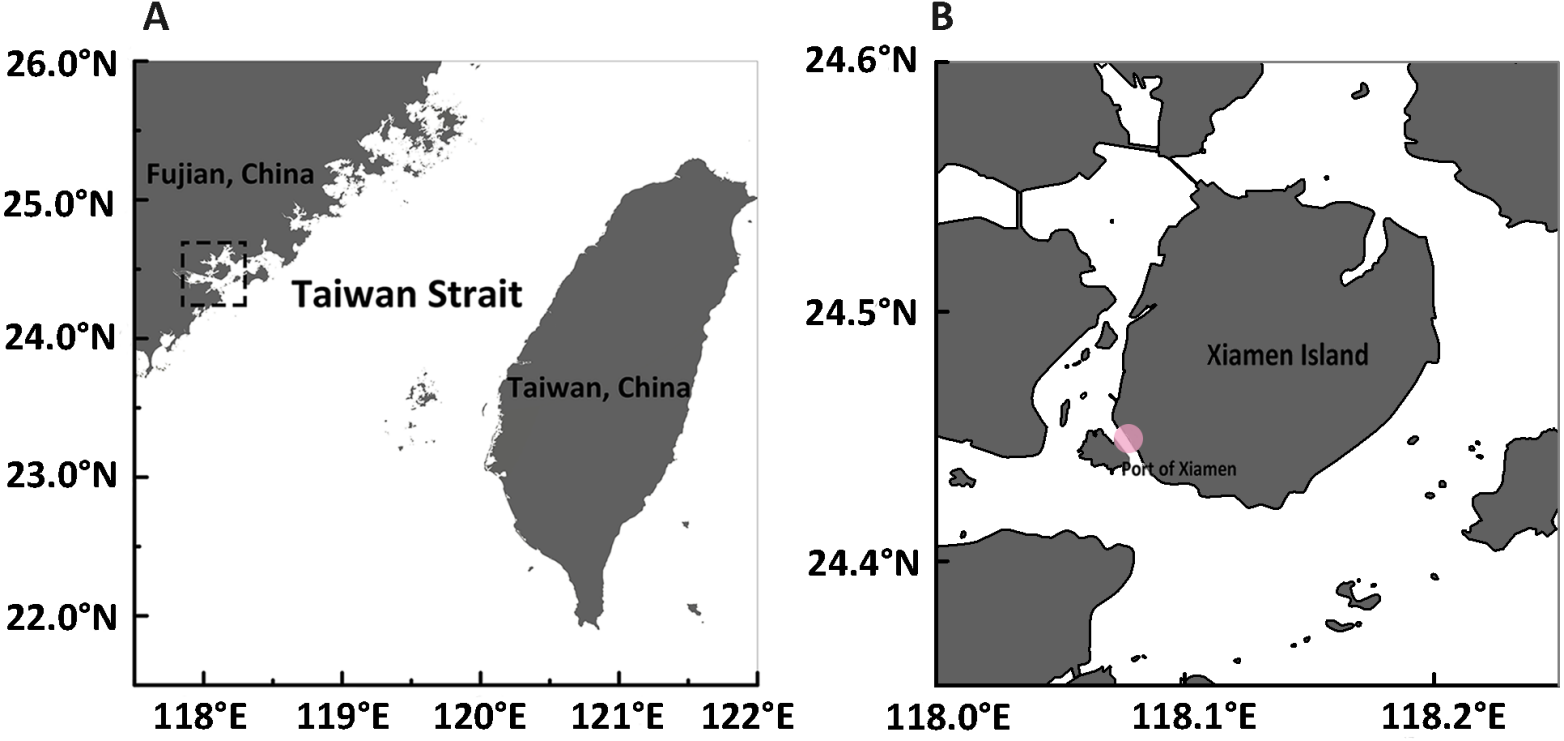
Maps of sampling site. (A) A panorama of Fujian and Taiwan beside both sides of the Taiwan Strait in southeastern China. The sample collection site is represented in the dotted rectangle. (B) The exact sample collection site at the edge of Xiamen Island is marked with a pink dot.

## Materials & Methods

### Sample collection and morphological observation

Samples were collected from Xiamen (E 118°04′, N 24°27′), China, in September of 2015 (Fig. 1) by trawling with a plankton net (505 µm mesh size, 0.8 m diameter) in shallow water during ebb tide (approval number for access to field sites is XDB06010103 from the Chinese Academy of Science). After collection, medusae were maintained in the laboratory in filtered seawater at room temperature and fed brine shrimp newly hatched from dry cysts (Qicaiyu, AIJIA PET AQUARIUM CO., LTD). The seawater was replaced every day by siphoning to clear egested debris and dead bait. For the reverse development assay, 108 medusae were injured by dissecting them into upper and lower halves (Fig. S1), as described by Piraino et al. (1996). Briefly, a scalpel was used to cut each medusa into two parts.The upper part consists of exumbrellar epidermis, mesoglea, subumbrellar endoderm, manubrium and the proximal part of the radial canals; the lower part contains tentacles, tentacle bulbs, velum, the whole ring canal, and the distal part of radial canals with a small part of medusa umbrella (exumbrellar epidermis, subumbrellar endoderm, mesoglea) also included. Both the upper and lower parts were cultured in different wells (approximately 10 mlseawater in each well) of plastic petri dishes (Corning Inc., Corning, NY, USA). The reverse development process was observed everyday and the morphology of each stage was noted and photographed under a stereoscope (Leica M165FC; Leica, Wetzlar, Germany). The rates of reverse development were calculated from one stage to the next. Specimens used for DNA analysis were starved for two days to avoid contamination from undigested food, and preserved in 95% ethanol.

### DNA extraction, PCR, and sequencing

Total genomic DNA was prepared from the whole medusa preserved in 95% ethanol with TIANamp Marine Animals DNA Kit (TIANGEN, Beijing, China), according to the manufacturer’s instructions. Approximately 540 bp of 16S rDNA and ∼650 bp of *COI* (cytochrome *c* oxidase subunit I) were amplified by Polymerase Chain Reaction (PCR). For the 16S rDNA partial sequence, the forward Primer was GAC TGT TTA CCA AAA ACA TA and the reverse primer was CAT AAT TCA ACA TCG AGG (Ender & Schierwater, 2003). The *COI* fragment was amplified using the forward primer TAA ACT TCA GGG TGA CCA AAA AAT CA and the reverse primer GTC AAC AAA TCA TAA AGA TAT TGG (Folmer et al., 1994). For both barcoding sequences, high fidelity PCR reactions were carried out using 15 to 30 ng of total genomic DNA as the template and set-up as follows: 2.0 µl of each primer (10 µM), 10.0 µl 5× buffer, 4.0 µl dNTP (each 10 mM), 1.0 µl PrimeSTAR HS DNA Polymerase (Takara, Shiga, Japan), and PCR-grade water to a total volume of 50 µl. The fragments of 16S rDNA were amplified with an initial denaturation step at 98 °C for 10 s followed by 30 cycles of 10 s at 98 °C, 15 s at 45 °C, and 30 s at 72 °C. Finally, there was a final extension for 5 min at 72 °C. For the *COI* PCR, the annealing temperature was increased to 50 °C, while other conditions remained unchanged. All PCR products were purified using TIANgel Midi Purification Kit (TIANGEN, Beijing, China) and sequenced in both directions on a 3730xl DNA Analyzer using BigDye BGI TECH SOLUTIONS (BEIJING LIUHE Co., Ltd, Beijing, China). Sequences were manually verified and the assembly of both strands was completed with DNAMAN v7. The partial 16S rDNA and *COI* sequences have been deposited in GenBank under accession numbers KY315334, KY315335, and KY315336 for 16S rDNA and KY315337, KY315338, and KY315339 for *COI*.

### Sequence alignment and phylogenetic analysis

16S rDNA and *COI* sequences were compared to 43 and 21 published sequences, respectively, from the *Turritopsis* genus (Table S1) from Genbank, among which IAAF01039897, IAAF01039898, IAAF01054602, and IAAF01054604 (*COI*) and IAAF01020395 (16S rDNA) were from the transcriptome data of *Turritopsis* in Japan (Hasegawa et al., 2016); the Bioproject number of the transcriptome is PRJDB4516. All of the sequences were aligned with ClustalW program in Mega 5.0 and edited manually in Editseq v7.1, in order to confirm the correct alignment and placement of insertion/deletion events. Three methods, Maximum Likelihood (ML), Maximum Parsimony (MP), and Neighbor Joining (NJ), were used to construct phylogenetic trees with Mega 5.0, using the Tamura 3-parameter + G model for 16S rDNA and the GTR + G model for *COI* as recommended by the built-in model test module. Node stability was assessed with 1,000 bootstrap replicates. Genetic distances within and between clades were computed using Mega 5.0 with the Kimura 2-Parameter mode for both 16S rDNA and *COI* sequences.

## Results

### Species identification

**Figure 2:**
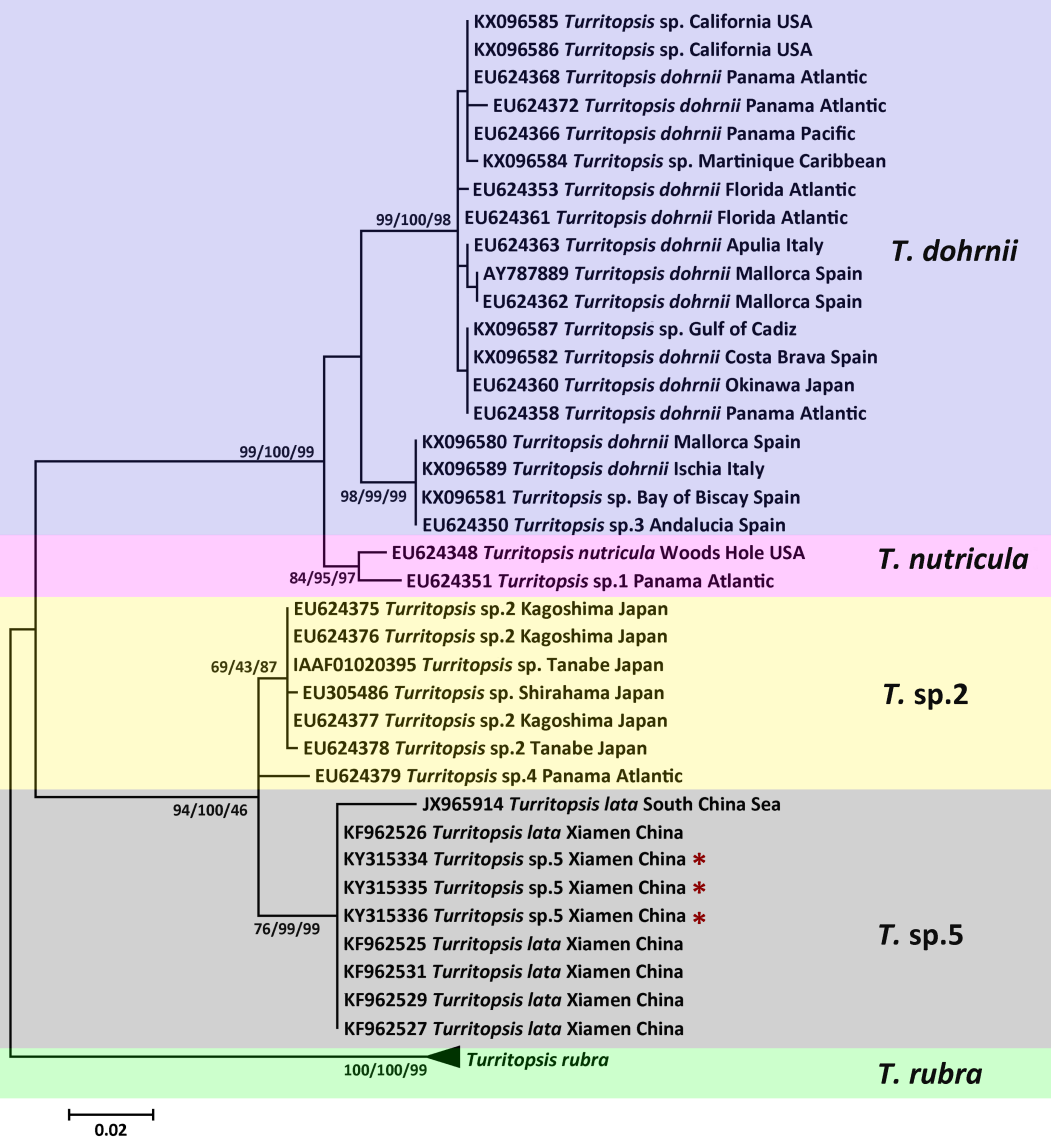
Phylogenetic analysis based on 16S rDNA partial sequences. Maximum Likelihood tree of the genus *Turritopsis* was based on mitochondrial 16S rDNA genes. A total of 46 16S rDNA sequences were analyzed. The *Turritopsis rubra* represents nine sequences of *T. rubra* 16S rDNA genes in Genbank (Table S1). For each node, Maximum Likelihood (the first number), Neighbor Joining (the second number), and Maximum Parsimony (the third number) bootstrap supports are reported (1,000 replicates). Bootstrap values were only shown if they were greater than 65% on the ML tree. The resulting clades are shown close to the branches. The scale bar represents 2% sequence divergence. The red asterisks denote the new sequences produced in this study.

**Figure 3:**
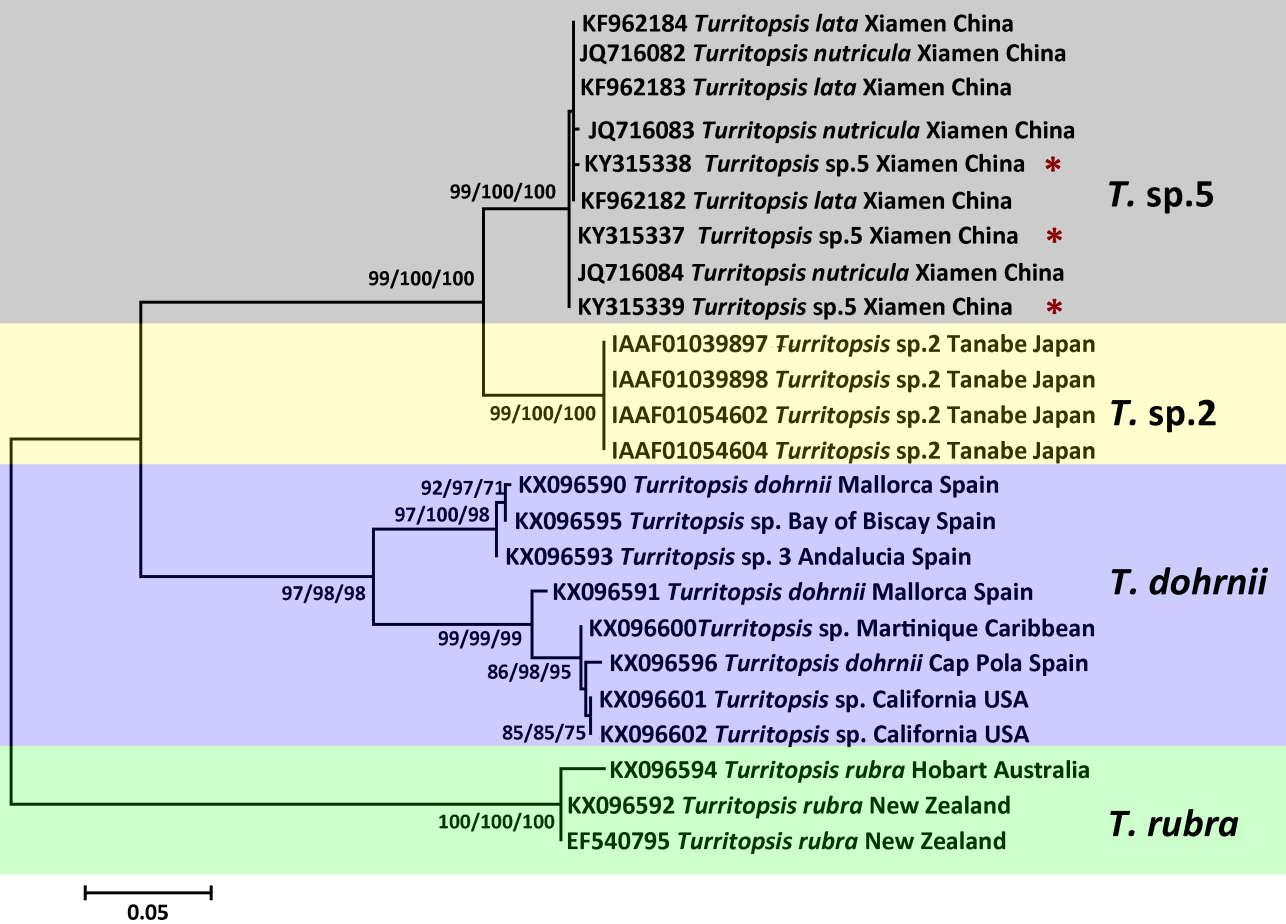
Phylogenetic analysis based on *COI* partial sequences. Maximum Likelihood tree of the genus *Turritopsis* based on the *COI* mitochondrial gene. A total of 24 *COI* sequences were analyzed. For each node, Maximum Likelihood (the first number), Neighbor Joining (the second number), and Maximum Parsimony (the third number) bootstrap supports are reported (1,000 replicates). Bootstrap values were only shown if they were greater than 65% on the ML tree. The resulting clades were shown close to the branches. The scale bar represents 5% sequence divergence. The red asterisks denote the new sequences produced in this study.

The topologies of trees resulting from ML, MP, and NJ analyses of 16S rDNA or *COI* sequences were consistent, and most bootstrap node values from all three analyses were similar, except for some of the Asian species on the 16S rDNA tree. The phylogenetic trees in Figs. 2 and 3 illustrate the five separate clades observed in the *Turritopsis* genus (note that there were no validated *COI* sequences for *T. nutricula* in Genbank, so only four clades were included in the *COI* tree), and this result acccords well with the tree constructed by Kubota (in press). Three of these have been designated as *T. rubra,* which is the most reliable and robust cluster in either tree and contains the barcoding information (EU624380, KX096592) from the type locality (Wellington Harbor, New Zealand) with a bootstrap value of 100%; *T. dohrnii*, a species comprising two distinct subclades, one of which was identified as *T.* sp.3 and mainly inhabits the western Mediterranean and along the southern European Atlantic coast, and the other has been assumed to be an invasive species worldwide (Miglietta & Lessios, 2009); *T. nutricula,* the clade has no available *COI* sequences, and the two 16S sequences representing the clade are not obtained from the type locality, therefore, the identity of this clade remains unconfirmed. Other unnamed clades are *T.* sp.2 and *T.* sp.5. *Turritopsis* sp.2 is a morphologically small form found in southern Japan with a high similarity to *T. dohrnii* from the Mediterranean Sea in life history and morphology (Miglietta et al., 2007). *Turritopsis* sp.4, found along the Atlantic coast of Panama, was also incorporated into this clade (Fig. 2), because the genetic distance between *T*.sp.4 and *T*.sp.2 (0.52%) was smaller than that between *T*.sp.4 and *T*.sp.5 (0.83%). *Turritopsis* sp.5 was the species we collected in Xiamen, China. The co-occurrence of some *T. nutricula* sequences (specimens collected in China) within the *T.* sp.5 clade in the tree based on *COI* might be interpreted as a possible misidentification. Considering that Schuchert classified *T. fascicularis* Fraser and *T. chevalense* Thornely as the same species in a closely related genus *Oceania* (Schuchert, 2016), and that he did not clearly address whether *Turritopsis* and *Oceania* had been combined into the same genus in his paper, these sequences were excluded from the phylogenetic tree.

An analysis of the Kimura 2-parameter distances within and between the five groups was performed, in order to further confirm the relationships between these clades (Table 1). The within-group divergence varied from 0.35% to 1.46% for 16S rDNA and from 0 to 6.70% for *COI*, while the genetic distances between our samples and other clades in the *Turritopsis* genus ranged from 3.07% (between *T.* sp.5 and *T.* sp.2) to 14.16% (between *T.* sp.5 and *T. rubra*) for 16S rDNA, and from 7.91% (between *T.* sp.5 and *T.* sp.2) to 24.51% (between *T.* sp.5 and *T. rubra*) for *COI*. This is a larger gap than the within-group distances for both genetic markers, consolidating the results that the species collected at Xiamen could form a clade distinct from the other four species in the *Turritopsis* genus.

**Table 1:** Interspecific and intraspecific variations in *Turritopsis* measured by Kimura 2-parameter distances (%) based on 16S rDNA and *COI* partial sequences.

	16S					*COI*			
	[1](%)	[2](%)	[3](%)	[4](%)	[5](%)	[1](%)	[2](%)	[3](%)	[4](%)
[1]*T*. sp.5(China)	0.35					0.15			
[2]*T*.sp.2(Japan)	3.07	0.6				7.91	0		
[3]*T. rubra*	14.16	10.44	0.37			24.51	23.85	1.01	
[4]*T. dohrnii*	13.71	10.8	11.45	1.46 (0^a^;0.40^b^)		20.78	20.89	22.21	6.70 (0.41^c^;1.36^d^)
[5]*T. nutricula*	11.63	10.24	11.58	4.01	1.42				

**Notes.**

a Intraspecific genetic distance of *T. dohrnii* European subclade based on 16S rDNA sequences.

b Intraspecific genetic distance of *T. dohrnii* worldwide subclade based on 16S rDNA sequences.

c Intraspecific genetic distance of *T. dohrnii* Europe subclade based on COI sequences.

d Intraspecific genetic distance of *T. dohrnii* worldwide subclade based on COI sequences.

**Figure 4:**
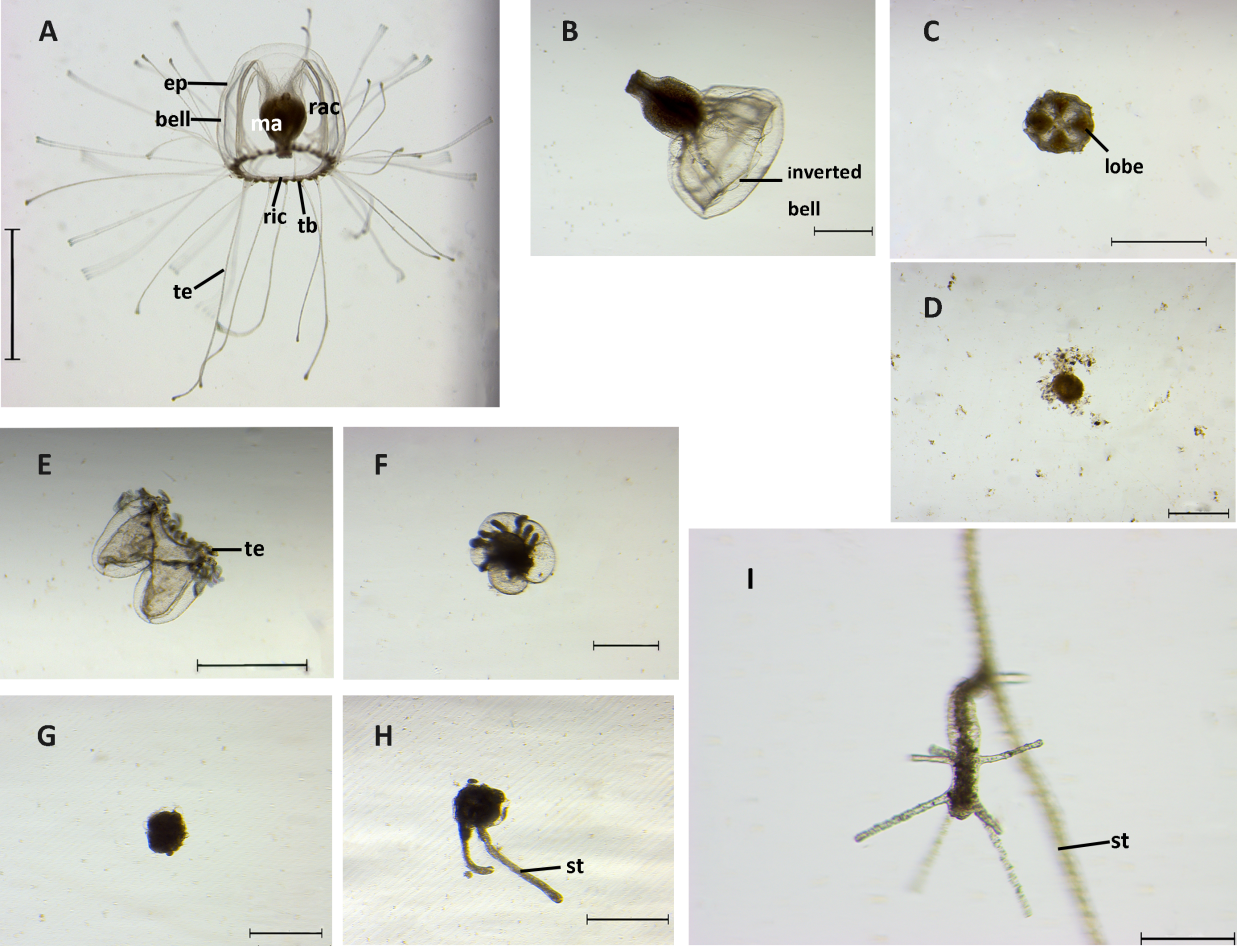
Stages of reverse development in *Turritopsis* sp.5 from Xiamen, China. (A) A mature medusa. Scale bar: 2 mm. (ep, epidermis; ma, manubrium; rac, radial canal; ric, ring canal; tb, tentacle bulb; te, tentacle) (B) Upper part of medusa after being cut with inverted bell. Scale bar: 500 µm. (C) Four-lobe clover shaped upper part cut from the medusa. Scale bar: 1 mm. (D) Degeneration of the upper part of the medusa after cut. Scale bar: 500 µm. (E) Contraction stage I: tentacles co ntracted after the medusa was cut. Scale bar: 1 mm. (te, tentacle) (F) Contraction stage II: lower part of medusa after cut began to fuse. Scale bar: 500 µm. (G) A ball-like cyst formed. Scale bar: 500 µm. (H) Two outgrowths of stolons. Scale bar: 500 µm. (st, stolon) (I) A rejuvenated polyp. Scale bar: 200 µm (st, stolon).

The morphology of *T.* sp.5 is illustrated in Fig. 4A which is described as follows: the mature medusa was bell-shaped with a flat to round top, and the overall measurements were 0.80–2.11 mm in height and 0.76–1.89 mm in width. The manubrium was on a gelatinous peduncle, the stomach was cruciform in the cross section and the mouth rim had four recurved lips. There were four broad radial canals and one ring canal, while the proximal parts of the radial canals had four blocks of vacuolated cells. Tentacle number ranged from 20–40 with adaxial red ocelli and inflated tentacle tips. The gonads were pale to light yellow on the interradial walls of the manubrium. Hydroids without medusa buds were monosiphonic in a stolonal colony, hydranths were of variable height, with up to 18 tentacles (12 on average) irregularly scattered over the distal part, and the hydrorhiza were reticular, coated with a transparent perisarc.

### Reverse development

The medusae displayed six distinguishable stages during the whole reversed life cycle, including medusa stage, contraction stage I, contraction stage II, cyst, cyst with stolon, and polyp. The morphologies of each stage were observed by stereoscope and are summarized in Fig. 4.

Medusae without injury had a bell-like umbrella with 20 to 40 long tentacles, and a canal system consisting of four radial canals originating from the base of the manubrium and a ring canal at the umbrella margin. With tentacles stretching from tentacle bulbs, they swam freely and actively (Fig. 4A). After the medusa was mechanically cut into two halves, the resultant two parts followed distinct developmental trajectories. The upper part of the medusa lost its ability to swim and most of them (around 90%) elongated their manubriums with the bell inverted (Fig. 4B); others attached to the substrate by the bottom of the manubrium and the interradial sectors of the bell shrank into a four-lobe clover-shaped structure (Fig. 4C). Nevertheless, even with both of these forms taken together, only a small proportion of the upper parts (2.78%) could completely develop into polyps, while the others completely degenerated within three weeks (Fig. 4D). The lower parts of medusae could still swim during the first two days after the cutting, but the tentacles gradually lost the ability to move and retracted around the ring canal. The original bell structure was still distinguishable. The above process was recognized as the contraction stage I, characterized by the tentacles being contracted (Fig. 4E). Two different states could then be observed. One was related to partial regeneration: some of the tissues in the lower part rehabilitated the damaged areas by regenerating the medusa bell structure and the tentacles then recovered from the contracted state to move slowly (Fig. S2). In the absence of a manubrium, this active state lasted a few weeks and then disintegrated. The other state was related to rejuvenation: the ring canal of the medusa fused together with parts of the tentacles and the sub-umbrella cavity shrank, which is the contraction stage II (Fig. 4F). The duration of contraction stages (I and II) in the lower part lasted up to 13 days, 75.93% of which went on to develop into a ball-like cyst. In the ball-like stage, fundamental medusa structures disappeared completely after merging into a ball-like structure, the “ball” of which was surrounded by a chitinous perisarc, forming a smooth surface and attaching to the substrate (Fig. 4G). Approximately one half to two days later, 47.56% of the “balls” developed stolons (whilst the others finally degenerated) (Fig. 4H). Finally, 43.59% of the creeping stolons produced polyps about two to five days after the stolon formed, and then continued living as a hydroid form (Fig. 4I). Overall, after three weeks of observation, 15.74% of medusae reverse developed into complete polyps and a large scale tissue degeneration was detected. Timetables and success rates of reverse development from one stage to the next are summarized in Table 2.

**Table 2:** Timetables and rejuvenation rates of the lower part of injured *Turritopsis* sp.5.

Stages	Duration time (days)	Rate of reverse development from one stage to the next (%)
Contraction–Cyst	2–13	75.93
Cyst–stolon	0.5–2	47.56
Stolon–polyp	2–5	43.59
Medusa–polyp	7–19	15.74

**Notes.**

The contraction stage I and II was not recorded separately, since the duration of contraction stage II was short. All tissues in the table originate from the lower part of medusa *T.* sp.5 after cut.

## Discussion

In order to circumvent the difficulty in the morphological classification of some tiny hydromedusae, which can be exacerbated by the plasticity of their morphological characteristics as a response to ambient conditions (Bouillon et al., 2006; Miglietta & Lessios, 2009; Miglietta et al., 2007), molecular approaches might be an effective and even indispensable means to determine real phylogenetic relationships between species in Hydrozoa. The mitochondrial 16S rDNA sequence is a useful genetic marker to allow for high resolution, and is routinely used for tackling difficult systematic questions at the species level in hydrozoans (Cunningham & Buss, 1993; Govindarajan, Halanych & Cunningham, 2005; Miglietta et al., 2007; Schuchert, 2005). In addition, since species barcoding is often done with *COI* sequencing, this locus was also used here as an additional marker. As seen in the phylogenetic trees that were based on both markers, sequences of our samples were clustered in the same clade as the sequences submitted by Zheng Lian-ming, who named the species *Turritopsis lata* von Lendenfeld. However, no definitive 16S sequence of *T. lata* has been submitted, and from a morphological viewpoint *T.* sp.5 differs from *T. lata* (Gershwin, Zeidler & Davie, 2010; Lendenfeld, 1885) in the following aspects: (1) mature *T*. sp.5 do not have an apical projection, but *T. lata* have a conical roof; (2) mature *T.* sp.5 are approximately 1.47 mm in height, which is much smaller than *T. lata*; (3) the maximal tentacular number of *T.* sp.5 is 40, which is less than that of *T. lata*; (4) the gonad color of *T*. sp.5 is pale to light yellow, however *T. lata* gonads are orange or brown. Therefore, the species we studied was tentatively named *T.* sp.5, following on from the serial numbers provided for other *Turritopsis* species by Miglietta & Lessios (2009).

While *T.* sp.2 from Japan also showed reverse development abilities, and was phylogenetically located close to the Chinese species *T.* sp.5 in both 16S and *COI* trees, the relationship between them and other species requires further investigation. When considering the barcoding data, no overlap was found between the inter-clade (3.07%–14.16% for 16S rDNA; 7.91%–24.51% for *COI*) and intra-clade (0.35%–1.46% for 16S rDNA; 0–6.70% for *COI*) Kimura 2-Parameter distances for *T.* sp.2 and *T.* sp.5. The genetic gaps would be larger if the intraspecific genetic distances were calculated with *T. dohrnii* split into two subclades (Table 1), and so it would be reasonable to identify *T.* sp.2 and *T*. sp.5 as two different species based on genetic information. However, the 16S sequence of *T*. sp.4, which was intermediate between the Chinese and Japanese species with an ambiguous affiliation and a geographically remote collection site (Panama Atlantic), might argue against the separation of *T.* sp.2 and *T*. sp.5 (Fig. 2). The strategy applied here was to tentatively affiliate the intermediate species into either of the two clades based on their genetic distances until more genetic information of the intermediates is available to clarify the relationship between the species of China and Japan. From a morphological perspective, the umbrella diameter of *T*. sp.5 from China (female 0.95–1.89 mm, male 0.76–1.51 mm) was marginally smaller than that of *T*. sp.2 from Japan (female 2.5–2.9 mm, male 2.0 mm, Kubota, 2005; 1.9–3.9 mm with female and male calculated together, Kubota, 2015). It should be noted, however, that there are insufficient morphological descriptions of the Japanese species, and considering the potential impact on such measurements and descriptions caused by morphological change in different developmental stages, both the Chinese and Japanese species require a more comprehensive description of their life histories in order to provide a clear delineation between these two species. Taking molecular and morphological evidences together, *T*. sp.5 in China can be separated from *T*. sp.2 in Japan to at least subspecies level, or even species level, but taking a conservative viewpoint one would not identify it as a new species since no unambiguous morphological evidence is available.

The ability to reverse the life cycle led to *Turritopsis* being awarded the moniker of the immortal jellyfish, nevertheless, the extent of this ability varies across different lineages of *Turritopsis*. *T. dohrnii* was demonstrated to rejuvenate into the polyp stage most easily in Piraino’s experiment with all young medusae transforming back into polyps under different stress conditions and all mature medusa transforming back spontaneously (Piraino et al., 1996; Schuchert, 2004). In contrast, sexual maturity would impose effects on rejuvenation rates of *T*. sp.2 (the small, southern *Turritopsis* population in Japan) with young medusa transforming much more easily into polyps than the subadult and the senile individuals if stressed or cultured in the same conditions (68.2% versus 9.1% for the young versus the subadult, Kubota, 2005; 83.3% versus 10.0% for the young versus the senile, Kubota, 2015). However, *T*. sp.2 still had a much higher rejuvenation rate (87.0%) than that of *T. rubra* (8.3%), the large medusa of the northern Japanese population, if reversals in both species were induced by needle sticking (Kubota, 2013). As for *T*. sp.5 in this study, although the statistics of the reversibility for *T*. sp.5 did not consider the mature and the young separately, the lower part of *T*. sp.5 definitely reverse the life cycle more easily than the upper part (15.9% versus 2.78%). These rates are clearly different from those in *T. dohrnii*, the reversibility rates of which for both the upper and the lower part ranged from 50% to 100% (Piraino et al., 1996). Overall, most clades in the tree (except *T.nutrucula*, about which there are no related reports) more or less show this trait. The variation between species could be related to their response to natural selection. If the ancestor of *Turritopsis* originated from the South Pacific (close to the habitats of the extant species *T.rubra*) and the trans-Arctic interchange contributed to dispersal of *Turritopsis* (Miglietta et al., 2007), the ability to reverse the life cycle could have been of adaptive value, helping to cope with extreme environments and surviving the migration into new habitats. Because environmental pressures would reinforce this trait to different extents in different locations after *Turritopsis* migrated from the South Pacific, the reversability would now be varied between different groups of *Turritopsis*.

Compared to *T. dohrnii*, not all tissues of *T. dohrnii* with the ability to reverse ontogenic direction could successfully reverse in *T.* sp.5. In *T. dohrnii*, manubriums together with cells from the exumbrellar epidermis and portions of the radial canal fully transformed into stolons and polyps, and so could the whole upper part of the medusa (Piraino et al., 1996). However, the upper part of *T.* sp.5, mainly consisting of exumbrellar epidermis, subumbrellar endoderm, manubrium, and radial canals, almost never formed polyps and eventually degenerated. The lower part of *T.* sp.5, containing tentacles, tentacle bulbs, the ring canal, and the exumbrellar epidermis could reverse develop into the young hydroid stage after mechanical injury, although at significantly lower rates than *T. dohrnii*. In summary, the ability of *T.* sp.5 to transform back into an earlier stage might be affected, not only by individual differences (degree of sexual maturity, difference in genetic background, etc.), but also by the different developmental competencies of distinct tissues. It is unknown whether this trait in *T.* sp.5 was inherited and only a small proportion of the offspring acquired it or, instead, it is regulated by a switch that turns off as the medusa gradually grow and mature. Either way, it would be interesting to determine the collaborative gene networks and the related metabolic pathways modulating the orientation of ontogeny. In order to provide a comprehensive understanding of the reverse development of *T.* sp.5, next generation sequencing technology should be applied in future studies (Hasegawa et al., 2016), and a comparison of gene expression between the upper part and the lower part of *T*. sp.5 after excision could be informative.

## Conclusions

*Turritopsis* sp.5 collected from Xiamen, China is separated from other *Turritopsis* species in phylogenetic trees based on both *COI* and 16S rDNA markers. This species shows the ability to reverse its life cycle if cut into two parts, each containing different tissue types. For the first time, the lower parts of the medusa show a higher efficiency of the reverse development process compared with the upper parts after mechanical injury. This phenomenon indicates that *Turritopsis* sp.5 has the potential to be used as an experimental model for reverse development.

##  Supplemental Information

10.7717/peerj.4225/supp-1 Figure S1 Dissection of *Turritopsis* sp.5 into two parts The dark blue line represents where the medusa was cut into halves. Scale bar: 2 mm.

10.7717/peerj.4225/supp-2 Figure S2 Regeneration of * Turritopsis* sp.5 after the upper part of the medusa was excised Regeneration of *Turritopsis* sp.5 after the upper part of the medusa was excised. Scale bar: 1 mm.

10.7717/peerj.4225/supp-3 Table S1 List of species in *Turritopsis* genus analyzed in this study

10.7717/peerj.4225/supp-4 Data S1 Raw data

## Additional Information and Declarations

### Field Study Permissions

The following information was supplied relating to field study approvals (i.e., approving body and any reference numbers): Samples were collected from Xiamen (E 118°04′, N 24°27′), China, in September of 2015 by trawling with a plankton net (505 µm mesh size, 0.8 m diameter) in shallow water during ebb tide (approval number for access to field sites is XDB06010103 from Chinese Academy of Science).

### DNA Deposition

The following information was supplied regarding the deposition of DNA sequences: GenBank: KY315334, KY315335, KY315336, KY315337, KY315338, KY315339.

### Data Availability

The following information was supplied regarding data availability: The raw data has been provided as a Supplemental File.
