# Supplementary figures and images for: Ontogeny reversal and phylogenetic analysis of Turritopsis sp.5 (Cnidaria, Hydrozoa, Oceaniidae), a possible new species endemic to Xiamen, China
Source: PeerJ. 2018 Jan 8;6:e4225. doi: 10.7717/peerj.4225 (PMC5764029; doi:10.7717/peerj.4225)

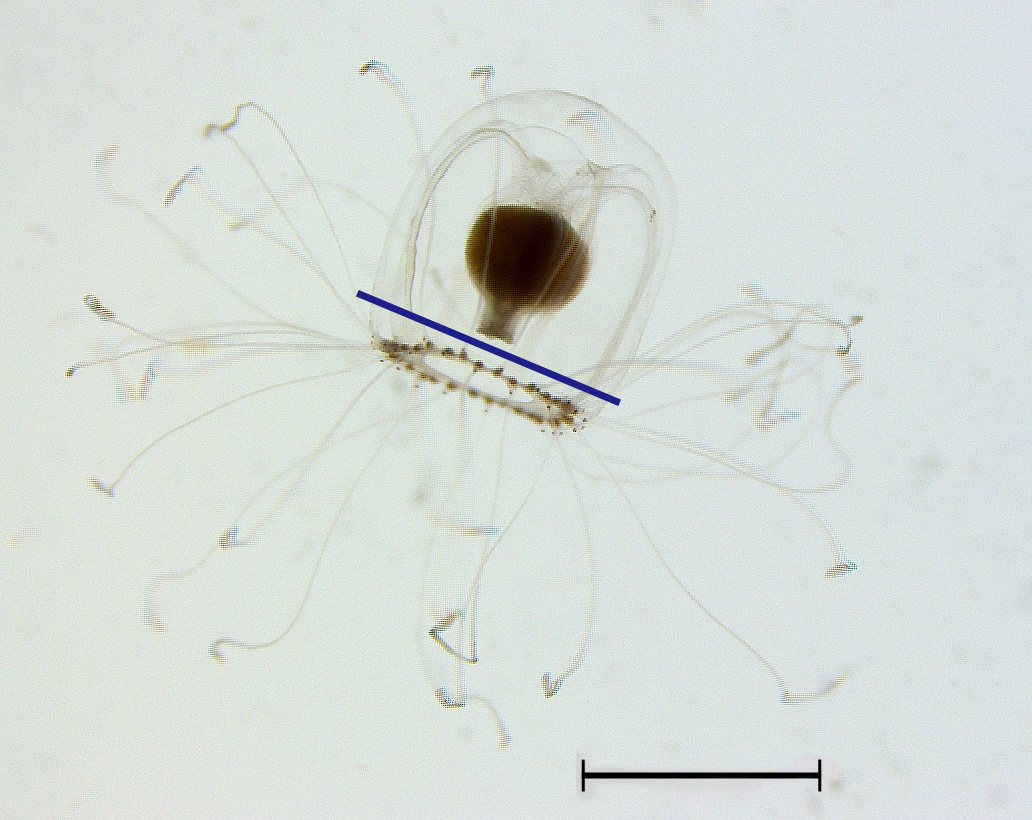

Supplement: Figure S1 — The dark blue line represents where the medusa was cut into halves. Scale bar: 2 mm. [file peerj-06-4225-s001.png]

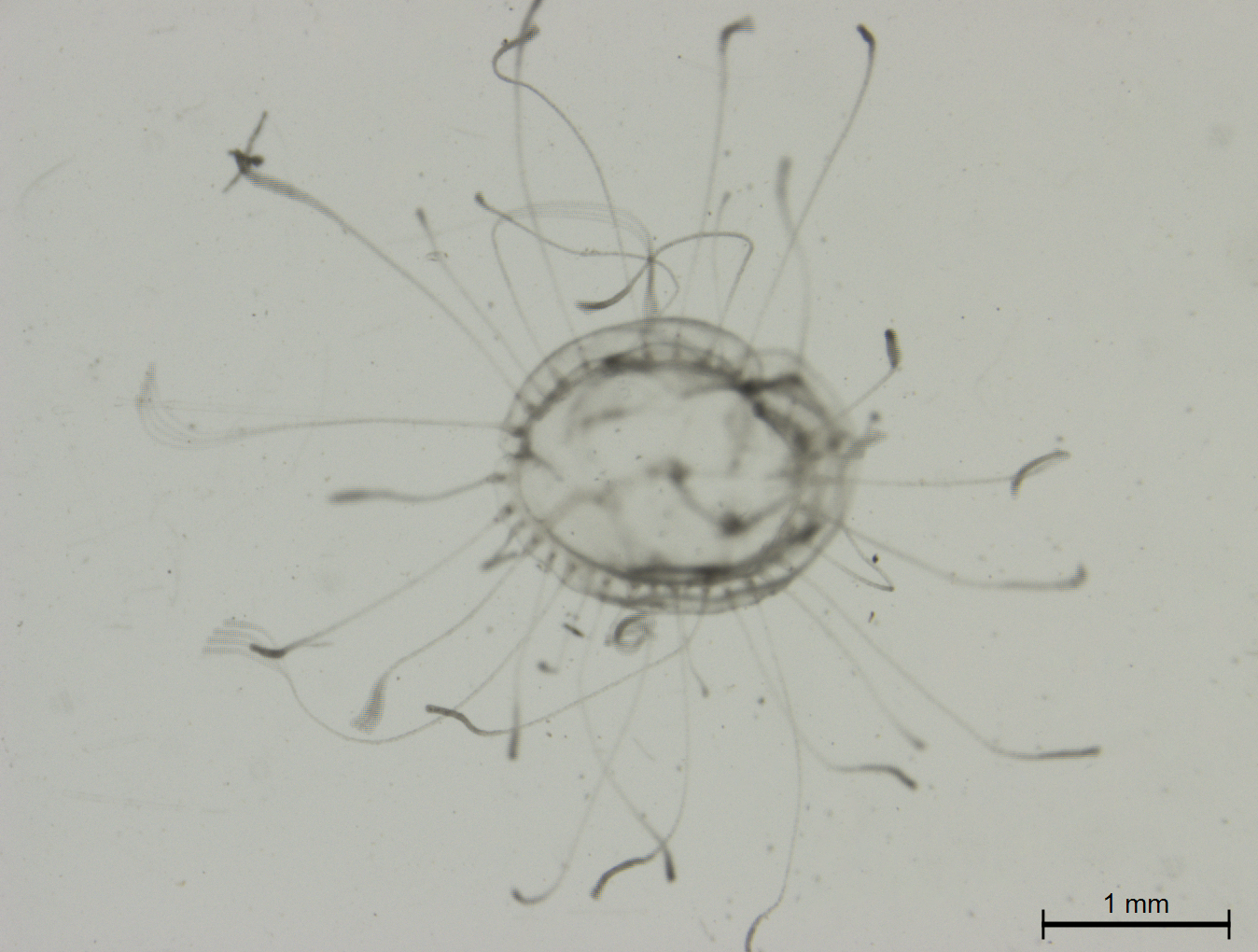

Supplement: Figure S2 — Regeneration of Turritopsis sp.5 after the upper part of the medusa was excised. Scale bar: 1 mm. [file peerj-06-4225-s002.png]
